# Antibody-Guided Therapy in Phospholipase A2 Receptor-Associated Membranous Nephropathy

**DOI:** 10.1016/j.ekir.2022.12.003

**Published:** 2022-12-13

**Authors:** Coralien H. Vink, Anne-Els van de Logt, Renate G. van der Molen, Julia M. Hofstra, Jack F.M. Wetzels

**Affiliations:** 1 Department of Nephrology, Radboud Institute of Health Sciences, Radboud University Medical Centre, Nijmegen, the Netherlands; 2 Department of Immunology, Radboud University Medical Centre, Nijmegen, the Netherlands; 3 Deptartment of Internal Medicine, Ziekenhuis Gelderse Vallei, Ede, the Netherlands

**Keywords:** anti-PLA2R antibodies, cyclophosphamide, membranous nephropathy

## Abstract

**Introduction:**

A 6-month course of cyclophosphamide (CP) and steroids is effective in primary membranous nephropathy (MN), but unappealing because of long-term side effects. We evaluated efficacy of an “antibody-guided” treatment schedule.

**Methods:**

Patients with phospholipase A2 receptor (PLA2R)-related MN and high risk of progression were treated with CP 1.5 mg/kg/d and steroids in cycles of 8 weeks. Anti-PLA2R antibodies were measured by indirect immunofluorescence (IIFT) at 8, 16, and 24 weeks, and a negative test resulted in withdrawal of CP, and rapid tapering of prednisone. In patients with persistent anti-PLA2R antibodies at 24 weeks, CP was switched to mycophenolate mofetil. Treatment was repeated in patients with a relapse.

**Results:**

Our analysis included 65 patients (48 males, 17 females), age 61 ± 12 years, estimated glomerular filtration rate (eGFR) 46 ml/min per 1.73 m^2^ (35−68), urine protein-to-creatinine ratio 7.7 grams/10 mmol creatinine (5.4−11.1) and serum albumin 20 g/l (16−26). Immunologic remission rate was 71% after 8 weeks, 86% after 16 weeks, 88% after 24 weeks, and 94% after 3 years. Twenty-seven patients (42%) had persistent clinical remission after only 8 weeks of therapy. Sixteen patients needed a second course of therapy because of immunologic or clinical relapse. Follow-up was 37 (26−58) months. Overall partial remission rate was 92%. One patient developed end-stage kidney disease. Antibody-guided therapy (ABG) was as effective as the standard 6-month course, whereas providing a lower cumulative dose of CP (11.1 [8.0−18.5] vs. 18.9 [14.2−23.6] grams).

**Conclusion:**

ABG is effective, and allows individualized therapy, with many patients responding to 8 weeks of CP-based therapy.

## Graphical abstract

**Figure:**
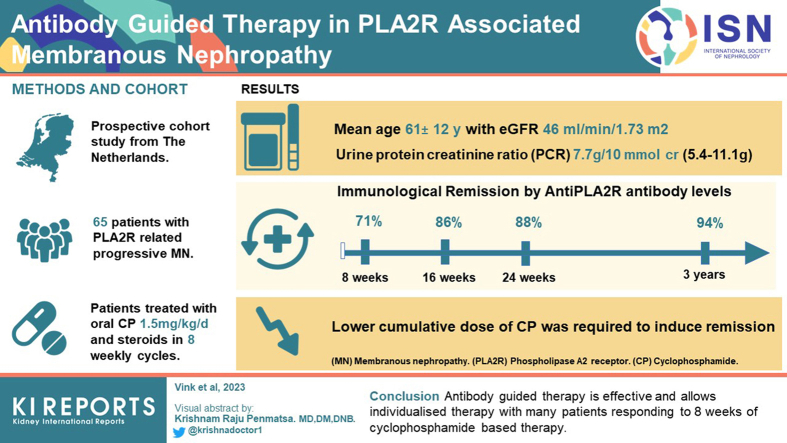

See Commentary on Page 397

Primary MN is one of the most common causes of nephrotic syndrome in adults.^1^ The clinical course is variable, with spontaneous remissions occurring in 30% to 40% of patients. Still, more than half of patients with MN and nephrotic syndrome may need immunosuppressive therapy.^2^^,^^3^ The 2012 Kidney Disease Improving Global Outcomes guideline recommended initial therapy with a 6-month course of alternating monthly cycles of prednisone and CP.^4^ This advice was on the basis of the evidence from randomized trials that demonstrated the efficacy of such therapy in preventing kidney failure in patients with MN at moderate-high risk.^5^^,^^6^ The original cyclical regimen uses a cumulative dose of 16 grams of CP in a typical 70 kg patient. We and others have administered daily CP for periods of 6 months to 12 months (cumulative dose of 19 g and 38 g, respectively) in high risk patients with similar success.^2^^,^^7^ Some authors have advocated a shorter treatment duration, providing a cumulative dose of 7 grams, although this was successful in low-risk patients only.^8^ Treatment with CP is criticized because of the dreaded short-term and long-term side effects. Because side-effects are related to the cumulative dose of CP, treatment dose and duration should be minimized, while maintaining efficacy.

The introduction of novel, less toxic immunosuppressive therapies has been advantageous. Still, the efficacy of calcineurin inhibitors and rituximab is debated. Calcineurin inhibitors induce remission, although complete clinical remissions are less frequent, immunologic remission rate is low in comparison to CP and rituximab, and relapse rate is high after drug withdrawal.^9^^,^^10^ Rituximab is more effective in inducing persistent remissions, still nonresponse is high at 35% to 40%.^9^ A recent cohort study reported almost 100% remission rate when using triple therapy consisting of rituximab, CP, and prednisone.^11^ Unfortunately, thus far, all suggested treatment protocols and trials use the “one size fits all” principle, exposing all patients to the same dose and duration of therapy. Optimization of therapy would benefit from personalized therapy.

In 2009, the identification of PLA2R1 as the major autoantigen in MN was a major breakthrough.^12^ It is now well established that in 70% to 80% of patients with MN, anti-PLA2R antibodies (aPLA2Rab) are present in the serum.^13^, ^14^, ^15^ Subsequent studies showed that measurement of aPLA2Rab provided clinically useful information. Beck *et al.*^16^ evaluated 35 patients, who were treated with rituximab. Changes of aPLA2Rab correlated with clinical outcome. Disappearance of aPLA2Rab preceded a clinical remission by 2 to 3 months.^16^ In another study, measurement of aPLA2Rab at the end of immunosuppressive therapy predicted long-term outcome as follows: 14 of the 24 patients in whom aPLA2Rab had disappeared were still in remission compared to none of 9 patients with persistent positive aPLA2Rab.^17^

The introduction of a sensitive IIFT assay allowed measurement of aPLA2Rab in clinical practice. We questioned if tailoring of CP therapy on the basis of aPLA2Rab-determined immunologic remission could be effective while allowing the use of a lower cumulative dose.

## Methods

This was a single-arm prospective cohort study, conducted in our academic center. The study protocol was approved by the regional ethical board (CMO regio Arnhem-Nijmegen). This study is registered at the Nederlands Trial Register (NL5890). We used STROBE criteria for the reporting of cohort studies (Supplementary Material).

In our region, many patients with MN are referred to our center for risk assessment as described.^18^ Patients with MN, either primarily seen at our outpatient clinic or referred from regional hospitals were considered for antibody-guided treatment.

Suitable for our study, as defined by the inclusion criteria, were adult patients (age >18 years) with MN, diagnosed by kidney biopsy or the presence of aPLA2Rab, and a high risk of disease progression. High risk was defined as persisting nephrotic syndrome for more than 6 months despite conservative treatment, and increased urinary excretion of beta-2-microglobulin (β2m) (>1000 ng/min) and/or increased excretion of alfa-1-microglobulin (α1m) (>40 μg/min),^19^ or deteriorating kidney function, or severe symptoms related to the nephrotic syndrome. Exclusion criteria were secondary MN, absence of aPLA2Rab, pregnancy, liver dysfunction, active infections, or participation in another study (STARMEN trial^10^). In case of a (relative) contraindication for treatment with CP (e.g., previous treatment [maximum cumulative exposure achieved] and/or comorbidity) patients were offered alternative treatment with mycophenolic acid and steroids or tacrolimus or rituximab.

Eligible patients were informed of the treatment protocol and provided informed consent for therapy, data collection, and analysis. Patients were managed in their local hospital. Patients and their physicians received the treatment protocol (see for details in the Supplementary Methods).

Immunosuppressive therapy consisted of intravenous methylprednisolone (1000 mg for 3 days at day 1−3, in some cases also at days 61−63 and 121−123), oral prednisone (0.5 mg/kg on alternate days) and oral CP 1.5 mg/kg/d. After 8, 16, and 24 weeks, serum was drawn for measurement of aPLA2Rab with an IIFT (commercial IIFT, EUROIMMUN Medizinische Labordiagnostika AG, Lübeck, Germany).^20^ If antibodies were negative, CP was withdrawn and prednisone was tapered in a period of 4 to 8 weeks (Supplementary Figure S1). Patients with persistent aPLA2Rab at 24 weeks were advised to continue treatment with mycophenolic acid (1000 mg twice daily) and low dose steroids, unless proteinuria was ≤1 g/d or ≤1 gram/10 mmol creatinine. In case of a clinical relapse after more than 12 months, patients were retreated with CP and steroids using the same strategy (with a maximum cumulative CP treatment duration of 6 months). Of note, patients with early relapse (≤12 months) or patients with persistent proteinuria and reoccurrence of aPLA2Rab were treated more extensively for at least the maximum allowed CP duration of 6 months (see Supplementary Methods).

To evaluate the efficacy of our antibody-guided treatment protocol, we compared the outcome of the current study with the outcome in historical controls treated with CP. These patients were treated from 1991 on with CP (1.5 mg/kg/d) and steroids.^21^ In the past, treatment duration was 12 months. More recently, we have used a treatment period of 6 months, which is more in agreement with the treatment duration as proposed by the often quoted “ponticelli” protocol.

All patients were advised to adhere to maximal conservative therapy as follows: a moderately salt-restricted diet, stringent blood pressure control (target 130/80 mm Hg), primarily by using angiotensin-converting enzyme inhibitors and/or angiotensin receptor blockers, and statins. Antiplatelet therapy was started when serum albumin levels (measured by bromocresol purple or immunonephelometric assay) dropped below 30 g/l, whereas if albumin levels dropped below 20 g/l a prophylactic dose of low molecular weight heparin was added, or antiplatelet therapy was discontinued and the patient was converted to a coumarin derivative.^22^

The primary outcome parameter was the cumulative incidence of clinical remission (partial- and complete remission) at 24 months, which had persisted for at least 6 months after the first course of ABG. The secondary end points included the incidence of immunologic remission, the incidence of overall clinical remission, the incidence of immunologic relapses (reoccurrence of aPLA2Rab) and clinical relapses, the duration of immunosuppressive treatment, the number of patients in need of additional immunosuppressive treatment, kidney function deterioration, and the occurrence of kidney failure and adverse events.

### Definitions and Calculations

For proteinuria data, 24-hour urine was used by preference; otherwise, a urine aliquot was used instead. To correct for inappropriate 24-hour urine collections, amount of proteinuria was expressed as protein-creatinine ratio (grams per 10 mmol of creatinine).

Nephrotic range proteinuria was defined as a protein-to-creatinine ratio ≥3.0 g/10 mmol creatinine. Complete clinical remission was defined as a protein-to-creatinine ratio ≤0.2 g/10 mmol creatinine with stable kidney function, and partial clinical remission was defined as protein-to-creatinine ratio <2.0 g/10 mmol creatinine with a reduction of >50% from baseline and stable kidney function. Achieving remission includes both partial and complete remission. Relapse was defined as proteinuria ≥3.0 g/10 mmol creatinine after prior clinical remission. An event was defined after repeated confirmatory measurements within 3 months (if available). Immunologic remission was defined as negative aPLA2rab according to IIFT test, and immunologic relapse was defined as positive IIFT test after a former negative test. In few patients, the IIFT was reported as ± for treatment decisions; according to protocol, this was considered as positive (no immunologic remission). We estimated GFR by applying the creatinine based 2009 “Chronic Kidney Disease Epidemiology Collaboration” equation. A decrease in eGFR of more than 40% after start of CP was considered as kidney function deterioration. An eGFR < 15 ml/min per 1.73 m^2^ was considered as kidney failure.

### Statistical Analysis

Data are presented as number (percent), means (± SD) or medians (interquartile ranges) when appropriate. *t*-test, Mann-Whitney *U* test, Kruskal Wallis test and Fisher exact test were used for comparisons between and within groups. The cumulative probability of a clinical event was estimated according to Kaplan-Meier analysis and evaluated using a log-rank test. All statistics were performed using IBM SPSS software, version 20 (Chicago, IL). Differences were considered significant with *P*-value < 0.05.

## Results

### Clinical Data

**Figure 1:**
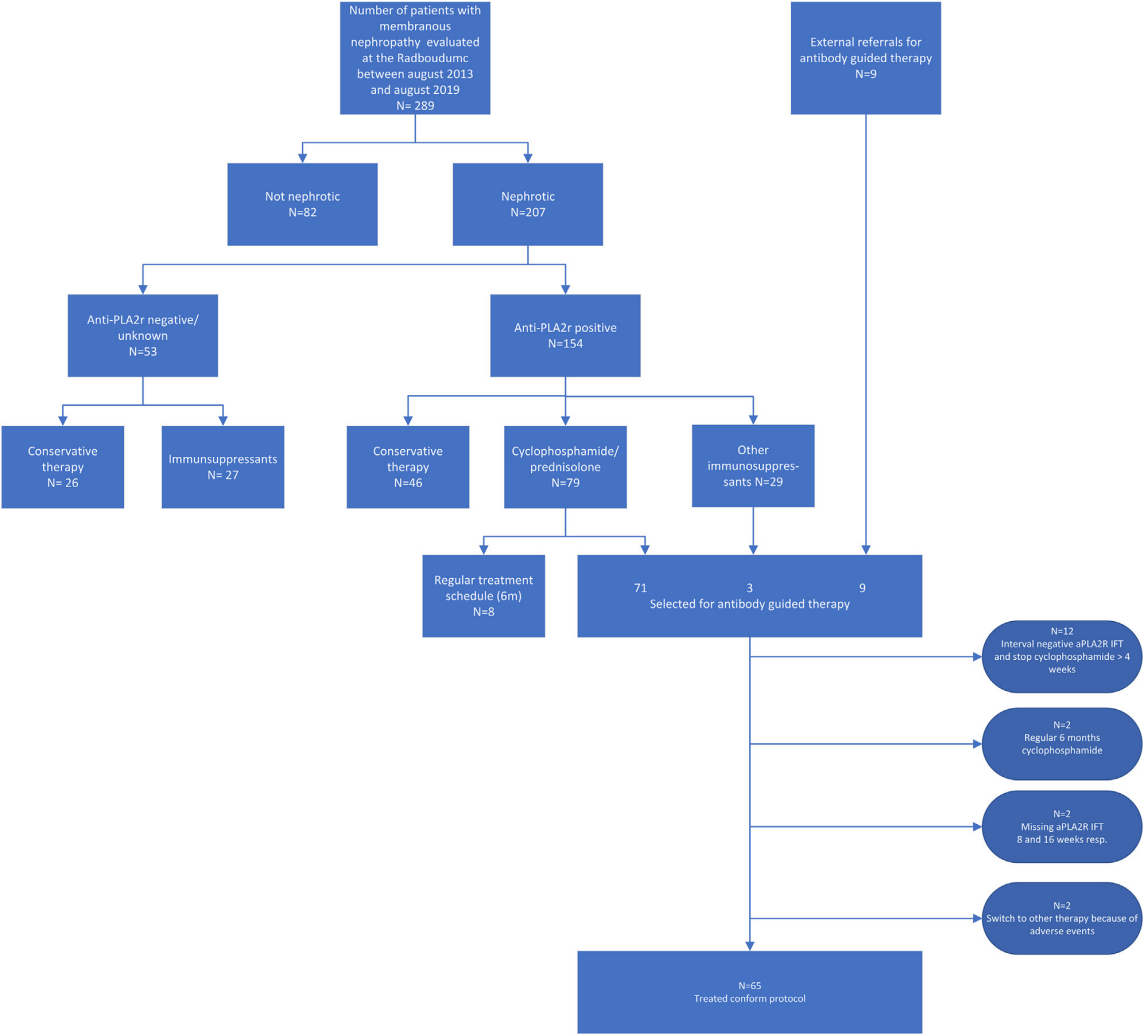
Flowchart of patients’ inclusion. PLA2R, phospholipase A2 receptor.

The study started in 2013. During the period from August 2013 to July 2019, 289 patients with MN were evaluated at the Radboud University Medical Center (Figure 1). Of these patients, 207 had a nephrotic syndrome, 154 of whom were aPLA2Rab positive. Of the 154 patients, 46 (30%) were treated with supportive therapy; 79 (51%) were advised CP and steroids, and of these, 71 patients were included in the study, whereas 8 received the standard 6-month regimen. In 29 patients, other immunosuppressants were initially started (tacrolimus ± steroids, mycophenolic acid + steroids, rituximab, high dose steroids started in referring center), of whom 3 (initially treated with tacrolimus ± steroids) eventually switched to ABG therapy with CP and steroids because of nonresponse. Nine patients were referred by other centers for inclusion in the study. Therefore, 83 patients were considered eligible and consented to the study protocol. For logistic reasons (missing serum samples, long delay between aPLA2Rab results and informing patients), physician and patients preference (preferring longer treatment duration), and side effects, 18 patients were not treated according protocol (Figure 1). Because our study specifically questioned the efficacy of short-term ABG, our analysis was restricted to patients treated according to protocol (*n* = 65).

Baseline characteristics of the excluded 18 patients were not different from the 65 patients treated according protocol. Clinical characteristics of the included patients (*n* = 65) are presented in Table 1. Kidney biopsy was performed in 60 patients and confirmed the diagnosis of MN in all of them. Mean age was 61 years, 74 % were male and 50 patients (77%) had new onset disease (incident patients), whereas 15 patients had prevalent disease, and were included after relapse (*n* = 12) or because of treatment resistance (*n* = 3). All patients were nephrotic, and eGFR was below 60 ml/min per 1.73 m^2^ in 42 patients (65%). The interval between diagnosis and start of immunosuppressive therapy was 5 (interquartile range 3−9) months in incident patients (*n* = 50) and 95 (interquartile range 31−142) months in patients with relapse or resistant disease (*n* = 15). Follow-up duration after start of antibody-guided CP therapy was 37 months (interquartile range 26−58).

**Table 1:** Clinical characteristics

Patients included in data analysis	65
Age (yr)	61 ± 12
Gender M/F (% male)	48/17 (74)
Time from diagnosis to therapy start (mo)	6 (3–30)
Diagnosis by biopsy	60 (92)
Patients with new onset disease	50 (77)
Patients after relapse	12 (19)
Previous spontaneous remission	5 (8)
Previous IS induced remission	7 (11)
Patients with treatment resistance	3 (5)
Baseline laboratory data	
Serum creatinine (umol/l)	136 (100–161)
eGFR by CKD-EPI formula (ml/min/1.73 m^2^)	46 (35–68)
Serum albumin (g/l)	20 (16–26)
Serum cholesterol (mmol/l)	6.4 (5.1–8.3)
Proteinuria (uPCR) (g/10 mmol)	7.7 (5.4–11.1)
ACE/ARB use	61 (94)

ACE, angiotensin-converting enzyme; ARB, angiotensin receptor blocker; CKD-EPI, chronic kidney disease-epidimiology collaboration; eGFR, estimated glomerular filtration rate; F, female; M, male; uPCR, urine protein:creatinine ratio.

Data presented as N (%), mean ± SD or median (IQR) according to variable type and distribution.

**Figure 2:**
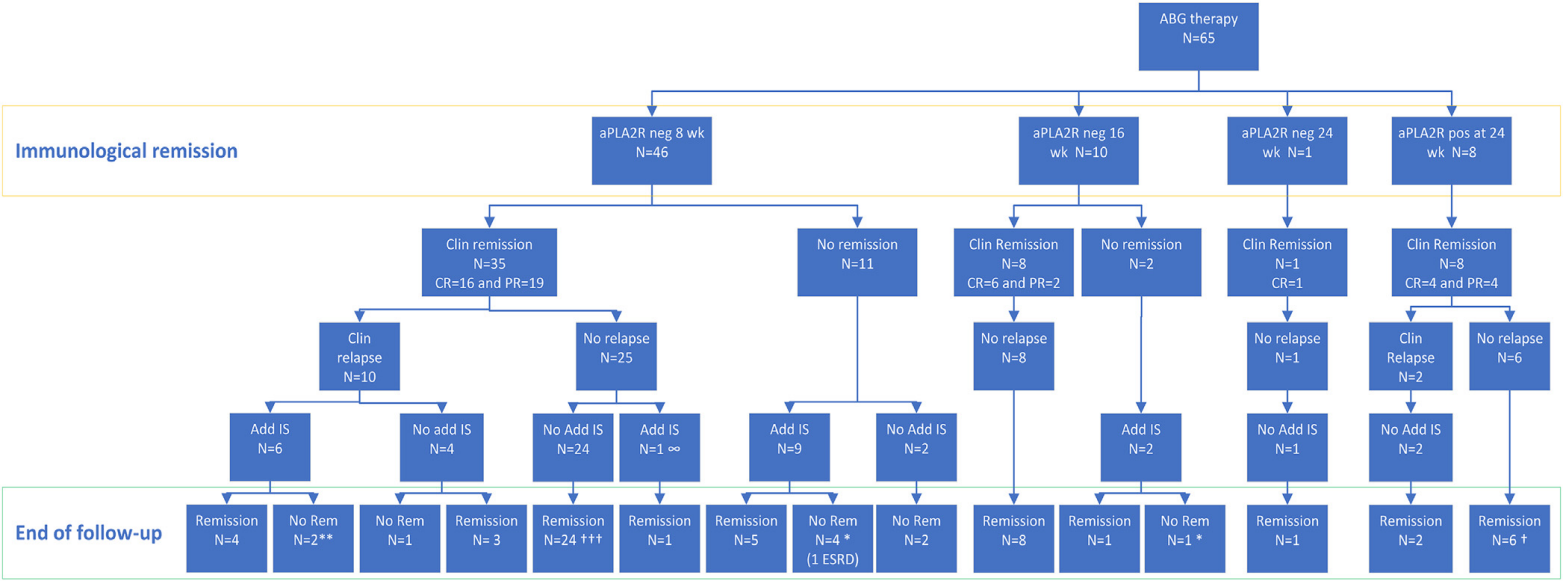
Flowchart of clinical events. In total, 52 remissions after the first course of ABG therapy are counted, of which 49 persisted >6 months. Each ∗ is 1 patient with short partial remission between additional immunosuppression and end of follow-up. ∞ in this patient additional IS was started because of slight increase in proteinuria and decrease in serum albumin, not fulfilling relapse criteria. Each † is 1 patient that died in remission. ABG, antibody-guided; aPLA2R, anti-PLA2R; CR, complete remission; ESRD, end-stage renal disease; IS, immunosuppressive therapy; PR, partial remission.

**Figure 3:**
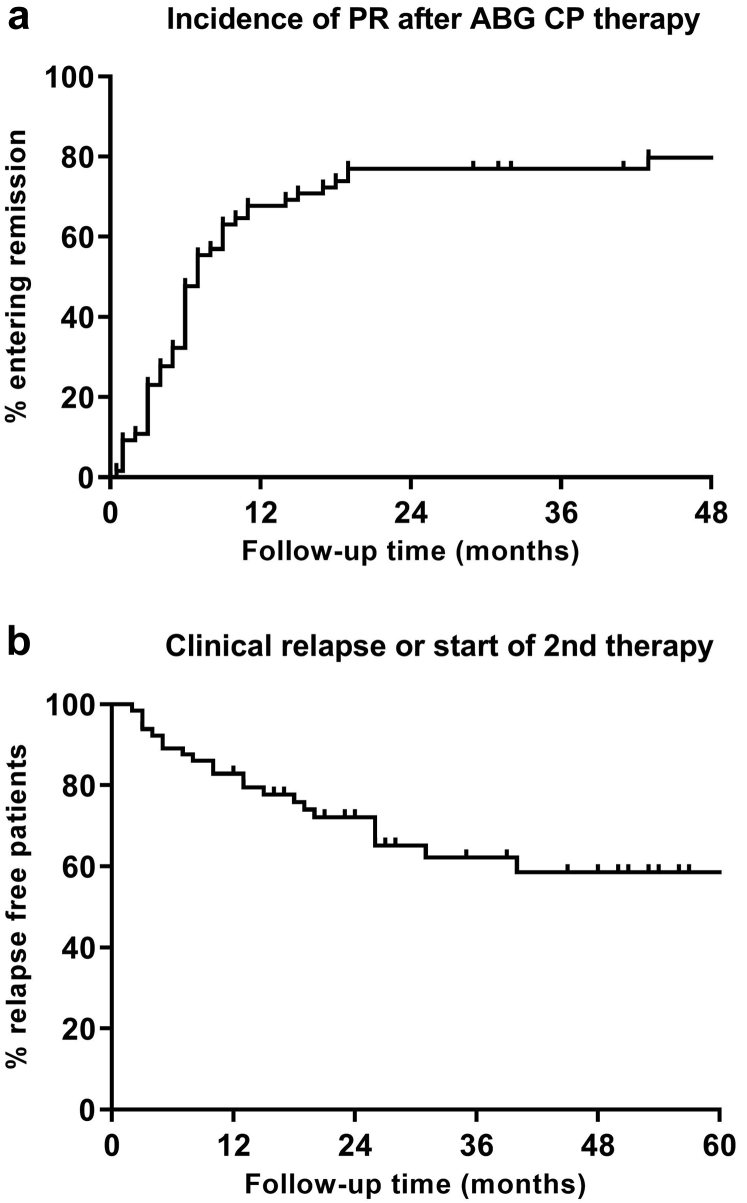
(a) Kaplan-Meier curves of clinical partial remission and (b) clinical relapse or second therapy.

Outcome data are presented in Table 2 and Figures 2 and 3. In 49 (75%) patients, the primary outcome criterium of clinical remission lasting for at least 6 months after the first course of antibody-guided treatment was met; in 27 (42%) patients, this was a complete remission.

**Table 2:** Outcome data

Immunologic outcome	

Cumulative incidence of immunologic remission (N (%))	
Wk 8	46 (71)
Wk 16	56 (86)
Wk 24	57 (88)
Mo 12	58 (89)
Mo 24	60 (92)
Mo 36	61 (94)
Clinical outcome	
Partial remission after first course of antibody-guided therapy (N [%])	52 (80)
Partial remission persisting for ≥6 mo (N [%])	49 (75)
Duration between start of therapy and PR (mo)	6 (3–9)
Complete remission after antibody-guided therapy (N [%])	27 (42)
Duration between start of therapy and CR (mo)	15 (11–22)
Clinical relapse after antibody-guided therapy (N [%])	12/52 (23)
Duration between PR and clinical relapse (mo)	15 (4–18)
Clinical partial remission after clinical relapse (N [%])	11/12 (92)
Overall clinical partial remission (after ABG ± additional courses of immunosuppressive therapy) (N [%])	60 (92)
Clinical partial remission at the end of follow-up (N [%])	55 (85)
Cumulative dosage of cyclophosphamide until the end of follow-up (grams)	11.1 (8.0–18.5) Min: 5.7 Max: 34.8
Follow-up duration (mo)	37 (26–58)
Kidney function deterioration/kidney failure (N [%])	1 (2)
Death during follow-up	4 (6)

ABG, antibody-guided; CR, complete remission; PR, partial remission.

Data presented as mean ± SD or median (IQR) according to variable type and distribution.

The cumulative incidence of immunologic remission was 71% at 8 weeks, 86% at 16 weeks, 88% at 24 weeks, 89% after 12 months, 92% after 24 months, and 94% at 36 months of follow-up. One patient developed immunologic remission after more than 3 years. In 8 patients, aPLA2Rab were still present after 24 weeks of therapy. Treatment was continued with mycophenolic acid in 6 of them and all 8 patients developed clinical remission (Figure 2). Notably, 3 patients did not develop immunologic remission during follow-up, although all 3 patients had good clinical response with partial or complete remission of proteinuria (apparent clinical-serologic dissociation, Supplementary Table S1A). Though immunologic remission was often followed by clinical remission (in 48 of 61 patients), persistent proteinuria was observed in 13 of 61 patients. In all but 2 patients with persistent proteinuria, an immunologic relapse was notable. Details of the patients with clinical-serologic dissociation are provided in Supplementary Table S1B. Ten patients with persistent proteinuria and antibody relapse received additional immunosuppressive therapy, leading to remission in 8 patients.

Overall, the cumulative rate of clinical remission was 92%. A clinical relapse occurred in 12 patients, which was accompanied by immunologic relapse in 9 patients. In 3 patients, there was no immunologic relapse, either because aPLA2Rab remained negative (*n* = 2) or had been persistently positive during clinical remission (Supplementary Table S1A and C). Eleven of 12 relapsing patients developed remission, either spontaneous (*n* = 5) or after renewed immunosuppressive therapy (*n* = 6).

At the end of follow-up, 55 patients were in clinical remission, 5 patients had proteinuria during relapse (of which 4 had recent relapse and were as yet untreated), and 5 patients had never achieved clinical remission, of which 1 developed kidney failure (Supplementary Table S2). Four patients died during follow-up, all who were in clinical remission. Clinical characteristics of the 5 patients with apparent clinical resistance are given in Supplementary Table S2. Three patients had achieved only temporary immunologic remission, with antibody relapse during follow-up. Thus these patients had persistent immunologic activity. In contrast, 2 patients had persistent immunologic remission. In the absence of repeated kidney biopsy, it remains unknown if the persistent proteinuria was caused by ongoing MN activity, or the consequence of glomerular injury related to secondary focal segmental glomerulosclerosis.

### Adverse Events

The adverse events are illustrated in Table 3. During the treatment and follow-up periods, 77 adverse events occurred in 39 patients (60% of total study population). Generally, one-third of adverse event were infections. There were 19 patients with a serious adverse event. Most occurred after withdrawal of therapy, and were considered unrelated to therapy. Two patients developed a malignancy; 1 lung cancer and 1 basal cell carcinoma of the skin, detected 43 months and 18 months after the start of CP, respectfully. The patient with lung cancer died of metastatic disease 1 month later, she was treated for 6 months with CP and for 7 months with mycophenolic acid, but also had a smoking history of 46 pack years. Three other patients died during follow-up of the study. One patient committed suicide 4 months after discontinuation of CP. One patient died because of extensive myocardial infarction 20 months after treatment with CP and being in partial remission with proteinuria of 0.5 g/d. The third patient died of necrotizing pancreatitis 44 months after the discontinuation of CP. During follow-up, 1 patient developed end-stage kidney disease.

**Table 3:** Adverse events

Adverse events	Total *N* = 65
Fever	3
Infection	26
Anemia	1
Leucopenia	8
Thrombopenia	1
Hyperglycemia	11
Liver toxicity	4
Malignancy	2
Minor cardiovascular events^a^	2
Major cardiovascular events^b^	6
Other^c^	13
ESRD	1
Deaths	4
Total number of adverse events	82
Number of severe adverse events^d^	19
Patients with adverse events (N [%])	42 (65)
Number of adverse events during or < 2 mo after ABG therapy	58
Infections during therapy	20
Follow-up duration after start of ABG therapy	37 [26–58]

ABG, antibody-guided; ESRD, end-stage renal disease.

a stable angina pectoris, peripheral vascular disease.

b myocardial infarction, stroke, amputation.

c Other AE include: sleeping problems (*n* = 1), depression (*n* = 1), sacrum fracture (*n* = 1), muscle weakness (*n* = 3), weight loss (*n* = 1), hair loss (*n* = 1), diarrhea and nausea (*n* = 2), severe edema (*n* = 1), neuropathy (*n* = 1) and hypogonadism (*n* = 1).

d Including hospitalizations, life threatening events, kidney failure, death during follow-up.

### Comparison With a Historical CP Treated Group

**Figure 4:**
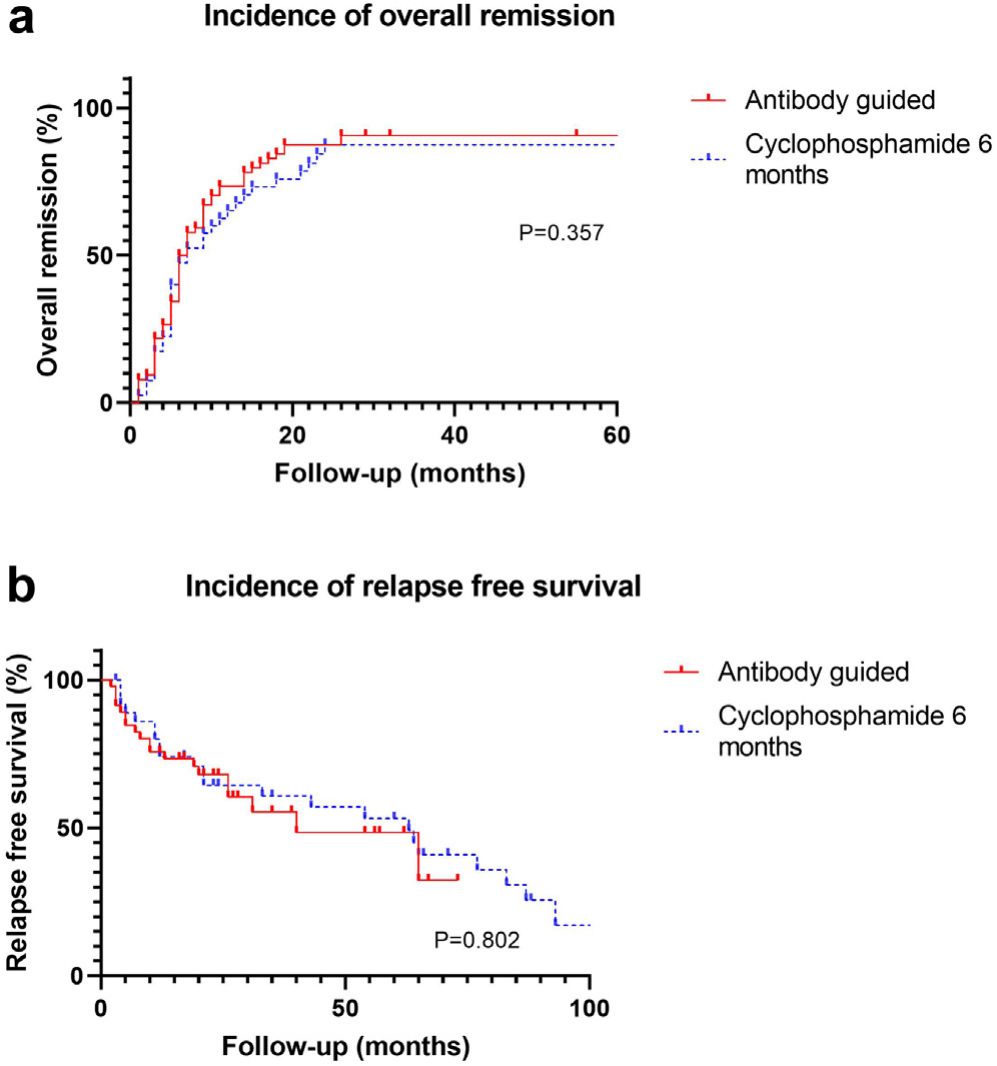
(a) Kaplan-Meier curves of overall clinical remission and (b) relapse free survival in patients with ABG therapy versus patients treated with cyclophosphamide during 6 months. Relapse was defined as relapse after clinical or immunologic remission.

For comparison, we selected a group of 40 patients treated with CP (1.5 mg/kg/d) for 6 months and steroids (Table 4). Information of 97 patients treated with CP during 12 months, nowadays considered unacceptable, is presented in Supplementary Figures S2 and S3). The cumulative dosage of CP was lower in the antibody-guided treatment cohort; with 11.1 (8.0−18.5) grams versus 18.9 (14.2−23.6) grams in the 6-month CP treated cohort. The overall rate of remissions was comparable (*P* = 0.357): 60 patients (92%) developed remission in the ABG-treated cohort versus 35 patients (88%) in the 6-month CP treated patients (Figure 4a).

**Table 4:** Clinical characteristics patients treated with cyclophosphamide during 6 months

Patients included in data analysis	40
Age (yr)	58 ± 11
Gender M/F (% male)	33/7 (83)
Time from diagnosis to therapy start (mo)	8 [2-16]
aPLA2R status	Positive: 26 (65)
	Negative: 11 (27)
	Inconclusive: 0 (0)
	Missing: 3 (8)
Diagnosis by biopsy	40 (100)
Patients with new onset disease	31 (78)
Patients after relapse	3 (7)
Previous spontaneous remission	1 (2)
Previous IS induced remission	2 (5)
Patients with treatment resistance	6 (15)
Baseline laboratory data	
Serum creatinine (umol/l)	125 (99–183)
eGFR by CKD-EPI formula (ml/min/1.73 m^2^)	53 (34–67)
Serum albumin (g/l)	21 (15–24)
Proteinuria (uPCR) (g/10 mmol)	8.1 (95.9–11.1)
ACE/ARB use	26/38 (68) 2 missing
Cumulative dosage of cyclophosphamide until the end of follow-up (grams)	18.9 (14.2–23.6) Min: 7.7 Max: 36
Follow-up duration (mo)	67 (27–95)

ACE, angiotensin-converting enzyme; aPLA2R, anti-PLA2R; ARB, angiotensin receptor blocker; CKD-EPI, chronic kidney disease-epidimiology collaboration; eGFR, estimated glomerular filtration; PLA2R, phospholipase A2 receptor.

Data presented as N(%), mean ± SD or median [IQR] according to variable type and distribution.

We also compared relapse rate. To prevent bias (some patients treated with ABG received treatment for a period of 6 months or more), we only included 46 patients initially treated according the ABG protocol for 8 weeks. Relapse was defined as relapse after clinical or immunologic remission. There was no difference in relapse rate (Figure 4b). Of note, we observed a significantly lower relapse rate in the 12-month CP treated cohort (Supplementary Figure S3).

## Discussion

Our study demonstrates that an individualized treatment protocol, using CP and steroids with treatment duration guided by regular monitoring of aPLA2Rab is effective. Overall, 94% of patients developed immunologic remission, 92% of patients developed clinical remission and only 1 of 65 patients developed kidney failure. These results were obtained using tailor-made therapy, with 42% of patients receiving only one 8-week course of CP. In addition, a significant number of patients (12%) had antibodies present and received treatment beyond 24 weeks, with apparent success. In a previous study, we showed that patients with persistent aPLA2Rab at the end of therapy did not develop sustained remission of proteinuria.^17^ Our current strategy, with treatment continued until remission (immunologic or clinical), showed high remission rates. Failure rate (patients not achieving clinical remission) was only 8%, and the latter patients either had immunologic relapse (and were not treated) or had persistent proteinuria while in immunologic remission, clinical features suggesting secondary focal segmental glomerulosclerosis. Therefore, monitoring of immunologic response during therapy contributes to improved, “tailor-made” therapy, with duration (and thus cumulative dose) of therapy adapted to the need of the patient.

It is well known that not all patients with MN and nephrotic syndrome need immunosuppressive therapy. At least 30% of patients will develop spontaneous remission, and these patients can be identified with reasonable accuracy with well-known biomarkers such as serum creatinine, urinary total protein, and urinary excretion of low molecular weight proteins.^18^ We regularly use these biomarkers in our restrictive treatment strategy, limiting the use of immunosuppressive therapy to patients at high risk of disease progression. The baseline clinical characteristics of the patients included in our study (and treated with CP and steroids) illustrate that our selection criteria perform well. Median proteinuria was 7.7 g/10 mmol, median serum albumin 20 g/l and eGFR was < 60 ml/min per 1.73 m^2^ in 65% of patients. It is evident that the risk profile of our patients was less favorable when compared to patients included in the recent STARMEN and RI-CYCLO trials.^10^^,^^23^

In the past decades, we have used CP-based therapy in patients with MN, on the basis of solid evidence from the pivotal randomized controlled trials that alkylating agents prevent kidney failure.^5^^,^^6^ The optimal duration of therapy with CP has been debated, with treatment duration (and cumulative dose) ranging from 3 months to 12 months (7 g−36 g). When comparing these studies, it appears that low dose therapy was used effectively only in low-risk patients, moderate dose therapy was used in moderate-high risk patients, whereas the high dose therapy was used in high-very high risk patients.^2^^,^^7^^,^^8^ Apparently, intensity of CP therapy was “intuitively” adapted to the severity of the disease. Our current study, which demonstrates that the required CP dose is variable, supports this interpretation.

To support our conclusions, we compared the data of the ABG-treated cohort with a historical cohort, treated with oral CP for 6 months. Outcome (remission rate and relapse rate) was comparable, whereas the cumulative dose of CP was significantly lower in the ABG-treated cohort (11 grams vs. 18 grams). Of note, we also evaluated a historical cohort of patients treated with CP for 12 months and noted a lower relapse rate in this cohort. This suggests that a higher cumulative CP dose might induce more stable remissions. Still, it is evident that approximately 5 patients need to be treated for 12 months to prevent 1 relapse. In view of the risks of therapy, and the treatability of a relapse, a shorter treatment course should be favored. Our new treatment protocol optimally balances risks and benefits.

Treatment with CP has been criticized for its toxicity. Newer treatment modalities have been proposed, including CNIs, rituximab, and a combination of them. Recent trials have provided evidence that these immunosuppressive agents induce immunologic and clinical remission.^10^^,^^23^, ^24^, ^25^ Still, the efficacy of these agents on hard renal end points has not been proven, the use of CNI monotherapy is associated with very high relapse rate after withdrawal, and in the recent trials of rituximab in MN, 35% to 40% of patients did not respond, even when they used a cumulative dose of rituximab of 4 grams.^9^^,^^24^^,^^25^ These patients might benefit from more intensive therapy. The benefit of intensive therapy is supported by the recent study of Zonozi *et al.*^11^ This study included 60 patients with MN. Treatment consisted of rituximab (8 doses of 1000 mg in a period of 24 months), CP (orally, 8weeks, 1.5 mg/kg/d) and prednisolone (treatment duration >16 weeks, cumulative dose ∼2.5 g). This study showed that with this long-term, high intensity therapy, MN can be very effectively treated, with remission rate of almost 100%. Of note, all patients received this intensive therapy. Our study indicates that we should abandon such “one size fits all” strategy, and we suggest that an antibody-guided treatment approach could also be advantageous when using the Zonozi *et al.*^11^ schedule, resulting in a shorter treatment duration and likely fewer side effects in up to two-thirds of patients.

Though monitoring for immunologic remission is an important step forward in improving MN treatment, our data indicate that reaching immunologic remission is not always followed by clinical remission. Specifically, of 46 patients with immunologic remission after 8 weeks of therapy, more than a quarter (11 of 46) did not develop clinical remission. In almost all patients, aPLA2Rab reappeared. Therefore, disappearance of antibodies is not sufficient to claim “immune tolerance.” However, majority of patients with immunologic remission reached and maintained clinical remission during follow-up. There were few patients with apparent “clinical-serological dissociation,” for which there is as yet no proven explanation. Although rare, these findings suggest that in the treatment of patients with MN, we should not solely rely on antibody responses.

Our study has limitations. We used CP, which has been the cornerstone of treatment in our center for many decades. In view of the recent trials, the use of CP can be limited, although a comparison of the outcome in the recent studies suggests that the best outcome is provided with therapy that contains 8 weeks of CP. Future studies should evaluate antibody-guided approach using different (combinations and sequence of) agents. The introduction of ABG therapy has implications for clinical trials; it should be debated if it is still acceptable to perform trials that compare treatment modalities based on the “one size fits all” principle. We question such approach.

The study was not randomized. We excluded patients who were not treated according to protocol. Most of the excluded patients continued treatment beyond 8 weeks despite immunologic remission, and inclusion of these patients would have biased (and favored) 8 weeks therapy. Therefore, we analyzed the data using a modified intention-to-treat principle, including patients who received the intended therapy. Of note, 2 patients were excluded because of early side effects (1 patient with pancreatitis because of pulse methylprednisolone, 1 patient because of liver toxicity). Inclusion of these patients would not have changed the conclusions. Another limitation is the use of only IIFT to measure aPLA2Rab. Several studies showed that the quantitative enzyme-linked immunosorbent assay methods has a lower sensitivity in detecting aPLA2Rab. A recent study suggested a new chemiluminescence assay as an alternative, better quantitative assay.^26^^,^^27^ The usefulness of these techniques for monitoring treatment should be considered.

In conclusion, antibody-guided treatment with CP and prednisone is effective and safe, with 42% of patients developing long-term clinical remission after one 8-week course of CP + prednisone therapy. More than 90% of patients developed clinical remission, underlining the efficacy of this treatment strategy. Still, in approximately 25% of patients, immunologic remission was not followed by clinical remission, underlining the need for predictive biomarkers. Antibody-guided therapy based on other less toxic agents should be tested in future trials.

## Disclosure

AL, CV and JW acknowledge support from a grant from the Dutch Kidney Foundation (grant nr NSN 17PhD12). All other authors have no competing interests.

## Supplementary Material

**Supplementary Methods.**

Concise methods.

**Figure S1.** Treatment schedule.

**Figure S2.** Kaplan-Meier curve of overall clinical remission in patients with ABG therapy versus patients treated with CP during 6 months and 12 months.

**Figure S3.** Kaplan-Meier curve of relapse free survival in patients with ABG therapy versus patients treated with CP during 6-months and 12 months. Relapse was defined as relapse after clinical or immunologic remission.

**Table S1.** Overview of patients with “clinical-serological dissociation.”

**Table S2.** Overview of patients without clinical remission.

**STROBE Statement.**
