# Supplementary material for: Antibody-Guided Therapy in Phospholipase A2 Receptor-Associated Membranous Nephropathy
Source: Kidney Int Rep. 2022 Dec 13;8(3):432–41. doi: 10.1016/j.ekir.2022.12.003 (PMC10014436; doi:10.1016/j.ekir.2022.12.003)
Supplement: Supplementary File (PDF) [file mmc1.pdf]

Supplementary appendix

Antibody guided therapy in patients with PLA2R-associated membranous nephropathy

CONCISE METHODS

Tapering of prednisone according to antibody guided regimen

Therapy= 0.5 mg/kg prednisone on alternate days during active treatment Week 1: 0.25 mg/kg on alternate days

Week 2: 0.15 mg/kg on alternate days

Week 3: 0.1 mg/kg on alternate days Week 4: 0.05 mg/kg on alternate days

If a patients is treated during 16 to 24 weeks the tapering is done according to the same regimen, however with intervals of two weeks' time.

Relapses after antibody guided CP/prednisone regimen

-If there was a need for additional immunosuppressive therapy within 12 months after start of antibody guided CP/prednisone (because of a relapse, or because of persisting proteinuria with a relapse of aPLA2Rab) the residual of the 6-months CP course was given (with a maximum of 6 months of CP cumulative).

-If there was a need for additional immunosuppressive therapy more than 12 months after the initial antibody guided CP course, another antibody guided CP course was given (with a maximum of 6 months of CP cumulative).

-In total patients could receive 3 antibody guided courses of CP, with a maximum of 6 months of CP cumulative.

- If additional immunosuppressive therapy was needed, but there was a (relative) contraindication for cyclophosphamide, patients were offered treatment with mycophenolic acid, tacrolimus or rituximab.

Measurements

Measurement of serum albumin: Our laboratory data are collected from multiple regional hospitals. Usually, the patients in our membranous nephropathy cohort are sent to the Radboudumc for second opinion / therapy advice, at this visit we ask for informed consent for collect follow-up data. Follow-up and if necessary immunosuppressive therapy is administered in the regional hospitals. Since the accuracy of serum albumin measurement in patients with nephrotic syndrome is very variable depending on the used assay (1) and not standardized, we do not take serum albumin level into account in our definitions.

1. van de Logt AE, Rijpma SR, Vink CH, Prudon-Rosmulder E, Wetzels JF, van Berkel M. The bias between different albumin assays may affect clinical decision-making. Kidney Int. 2019;95(6):1514-7.

STROBE Statement—Checklist of items that should be included in reports of *cohort studies*

	Item No	Recommendation	Page No
**Title and abstract**	1	(*a*) Indicate the study's design with a commonly used term in the title or the abstract	P1
		(*b*) Provide in the abstract an informative and balanced summary of what was done and what was found	P2
		**Introduction**	
Background/rationale	2	Explain the scientific background and rationale for the investigation being reported	P3,4
Objectives	3	State specific objectives, including any prespecified hypotheses	P4
**Methods**			
Study design	4	Present key elements of study design early in the paper	P4
Setting	5	Describe the setting, locations, and relevant dates, including periods of recruitment, exposure, follow-up, and data collection	P4,7
Participants	6	(a)Give the eligibility criteria, and the sources and methods of selection of participants. Describe methods of follow-up	P4,5
		(b)For matched studies, give matching criteria and number of exposed and unexposed	na
Variables	7	Clearly define all outcomes, exposures, predictors, potential confounders, and effect modifiers. Give diagnostic criteria, if applicable	P6
Data sources/	8*	For each variable of interest, give sources of data and details of methods of	P6,7

measurement		assessment (measurement). Describe comparability of assessment methods if there is more than one group	
Bias	9	Describe any efforts to address potential sources of bias	P4
Study size	10	Explain how the study size was arrived at	na
Quantitative variables	11	Explain how quantitative variables were handled in the analyses. If applicable, describe which groupings were chosen and why	P6,7
Statistical methods	12	(*a*) Describe all statistical methods, including those used to control for confounding<br>(b) Describe any methods used to examine subgroups and interactions<br>(c) Explain how missing data were addressed<br>(d) If applicable, explain how loss to follow-up was addressed<br>(e) Describe any sensitivity analyses	P7
**Results**			
Participants	13*	(a) Report numbers of individuals at each stage of study—eg numbers potentially eligible, examined for eligibility, confirmed eligible, included in the study, completing follow-up, and analysed<br>(b) Give reasons for non-participation at each stage<br>(c) Consider use of a flow diagram	P7,8<br>Fig 1
Descriptive data	14*	(a) Give characteristics of study participants (eg demographic, clinical, social) and information on exposures and potential confounders<br>(b) Indicate number of participants with missing data for each variable of interest	P8<br>Table 1

		(c) Summarise follow-up time (eg, average and total amount)	
Outcome data	15*	Report numbers of outcome events or summary measures over time	P8- 12
Main results	16	(*a*) Give unadjusted estimates and, if applicable, confounder-adjusted estimates and their precision (eg, 95% confidence interval). Make clear which confounders were adjusted for and why they were included	P8- 12
	(b)	Report category boundaries when continuous variables were categorized	
	(c)	If relevant, consider translating estimates of relative risk into absolute risk for a meaningful time period	
Other analyses	17	Report other analyses done—eg analyses of subgroups and interactions, and sensitivity analyses	na
**Discussion**			
Key results	18	Summarise key results with reference to study objectives	P10
Limitations	19	Discuss limitations of the study, taking into account sources of potential bias or imprecision. Discuss both direction and magnitude of any potential bias	P11-12
Interpretation	20	Give a cautious overall interpretation of results considering objectives, limitations, multiplicity of analyses, results from similar studies, and other relevant evidence	P11-13
Generalisability	21	Discuss the generalisability (external validity) of the study results	P11-13
**Other information**			
Funding	22	Give the source of funding and the role of the funders for the present study and, if applicable, for the original study on which the present article is based	P14

Table S1: Overview of patients with 'clinical-serological dissociation' A: Patients with clinical remission with persistent aPLA2rab

Study number	Time from start of therapy until clinical remission	aPLA2rab status at remission	Continued therapy	Total follow- up duration	aPLA2rab status during follow-up	Clinical status at end of follow-up
13	Partial Remission after 2 months	IIFT 3+	MMF for 12 months	60 months	IIFT still 3+ 1 year after clinical partial remission, no samples afterwards	Complete remission after 13 months, lasting till end of follow-up, referred back to GP
30	Partial remission after 18 months	IIFT 3+	MMF until end of follow-up	34 months	ELISA titer 29 u/mL at end of follow-up	Partial remission (proteinuria 0.4g/day)
12	Partial remission after 1 month, complete remission after 21 months	IIFT 2+	MMF for 11 months	62 months	IIFT still 2+ for 37 months after clinical partial remission, long interval between samples, IIFT negative at last follow-up	Complete remission
44	Partial remission after 7 months	IIFT+	No	29 months	IIFT+ 1 year after clinical remission, no samples afterwards	Partial remission (in between a short period of clinical relapse, with spontaneous improvement)

B: Patients with persistent proteinuria without aPLA2rab relapse

Study number	Incident/ prevalent patient	Time to immunological remission	Antibody relapse	2nd therapy	Clinical remission	Follow-up duration (months)	End of follow-up
18	incident	8 weeks	No	-	No	65	~15% decrease in eGFR, proteinuria likely due to secondary FSGS (uPCR 3.3 g/10 mmol, serum albumin 43 g/L, serum creatinine 198 umol/L)
42	incident	8 weeks	No	4 months CP/pred	No	32	Stable kidney function, proteinuria likely due to secondary FSGS (uPCR 2.4 g/10 mmol, serum albumin 40 g/L, serum creatinine 125 umol/L)

C: Patients with proteinuria relapse, without antibody relapse

Study number	Incident/ prevalent patient	Time to immunological remission	Antibody remission	Time to clinical partial remission (months)	Clinical complete remission	Time from clinical remission to clinical relapse (months	Antibody relapse	2nd therapy	Follow-up duration (months)	PR at the end of follow-up
10	Incident	8 weeks	Yes	6	No	3	No	No	66	Yes
29	Incident	>24 weeks	Yes, after continued therapy	19	No	13	No	No	36	Yes

Table S2: Overview of patients without clinical remission

Study number	Incident/ prevalent patient	Time to immunological remission	Antibody relapse	2nd therapy	2nd antibody remission	Clinical remission	2nd antibody relapse	Follow-up duration (months)	End of follow-up
18	incident	8 weeks	No	-	-	No	-	65	~15% decrease in eGFR, proteinuria likely due to secondary FSGS (uPCR 3.3 g/10 mmol, serum albumin 43 g/L, serum creatinine 198 umol/L)
42	incident	8 weeks	No	4 months CP/pred	-	No	-	32	Stable kidney function, proteinuria likely due to secondary FSGS (uPCR 2.4 g/10 mmol, serum albumin 40 g/L, serum creatinine 125 umol/L)
20	prevalent	8 weeks	Yes	MMF/pred	No	No		55	ESRD
49	incident	8 weeks	Yes	-	No	No	-	29	Persisting aPLA2Rab, persisting proteinuria. Patient prefers conservative therapy
53	incident	8 weeks	Yes	4 months CP/pred	Yes	No	Yes	29	Stable kidney function, aggravated nephrotic syndrome, about to start tacrolimus

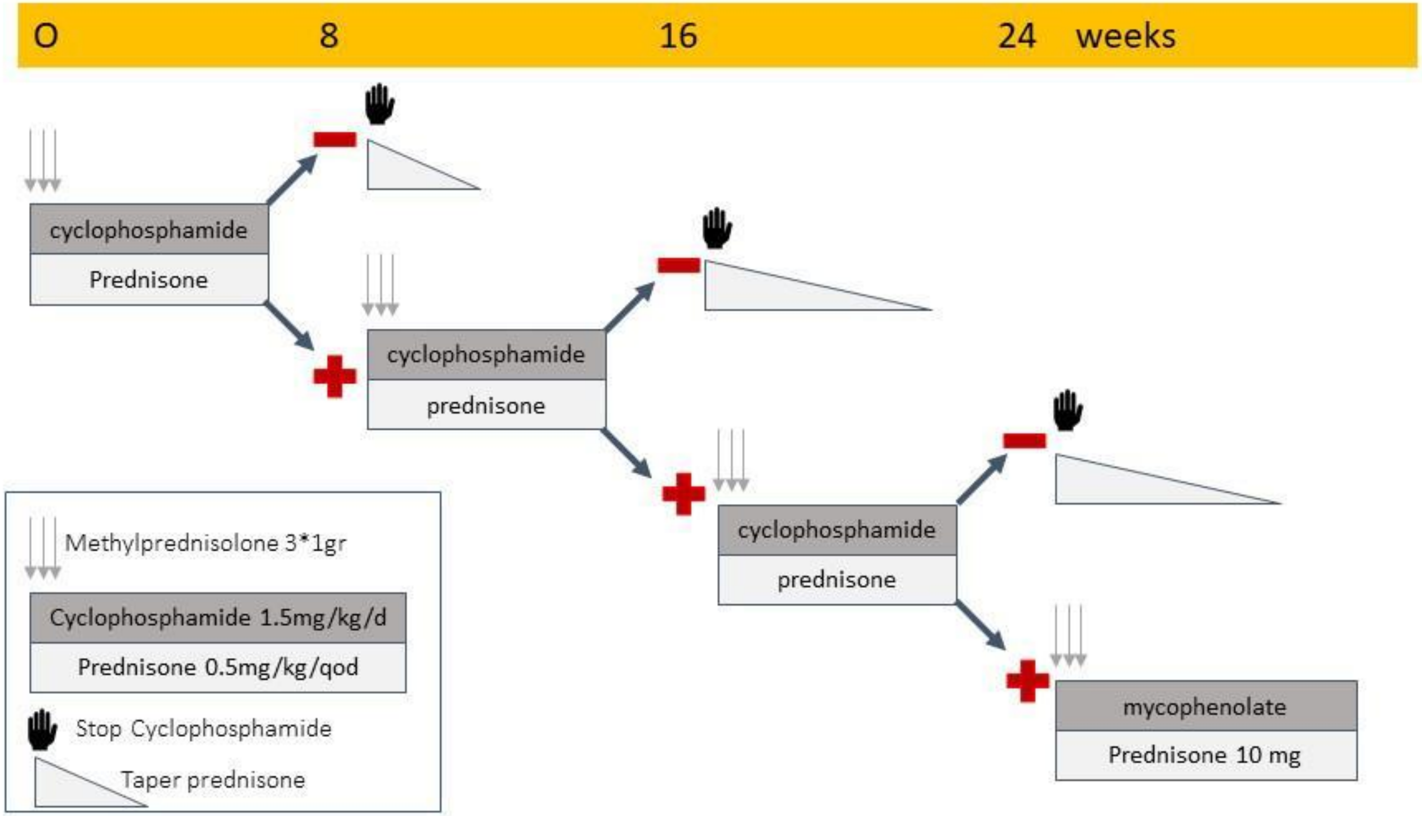

Figure S1: treatment schedule

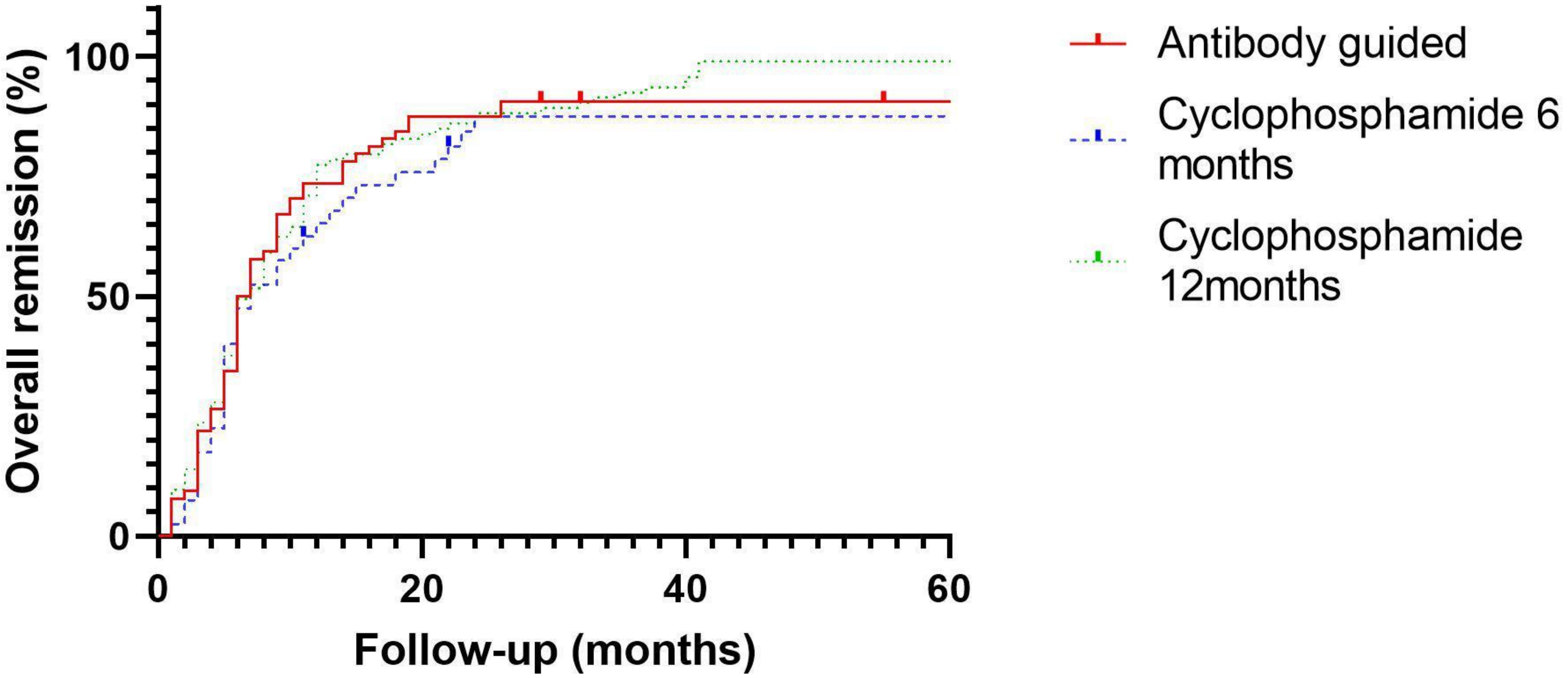

Figure S2**:** Kaplan Meier curve of overall clinical remission

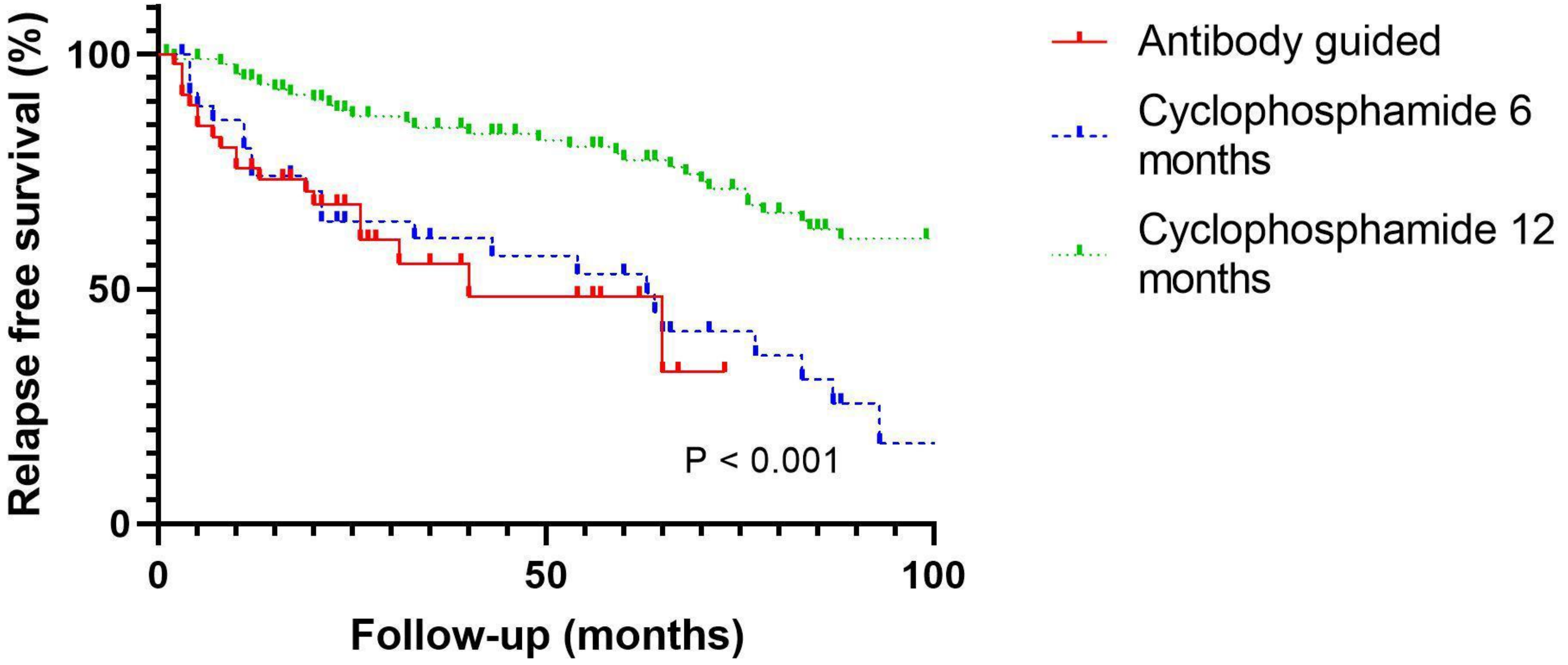

Figure S3**:** Kaplan Meier curve of relapse free survival
